# Iron content of glioblastoma tumours and role of ferrous iron in the hypoxic response *in vitro*

**DOI:** 10.3389/fonc.2025.1536549

**Published:** 2025-03-07

**Authors:** Citra Praditi, Eira Beverley-Stone, Malcolm Reid, Eleanor R. Burgess, Rebekah L. Crake, Margreet C.M. Vissers, Janice A. Royds, Tania L. Slatter, Gabi U. Dachs, Elisabeth Phillips

**Affiliations:** ^1^ Mackenzie Cancer Research Group, Department of Pathology and Biomedical Science, University of Otago Christchurch, Christchurch, New Zealand; ^2^ Centre for Trace Element Analysis, Department of Geology, University of Otago, Dunedin, New Zealand; ^3^ Department of Immunobiochemistry, Medical Faculty, Mannheim Institute for Innate Immunoscience (MI3), Heidelberg University, Mannheim, Germany; ^4^ Oncogenic Transcription Laboratory, Olivia Newton-John Cancer Research Institute, Melbourne, VIC, Australia; ^5^ Mātai Hāora, Centre for Redox Biology and Medicine, Department of Pathology and Biomedical Science, University of Otago, Christchurch, New Zealand; ^6^ Department of Pathology, Dunedin School of Medicine, University of Otago, Dunedin, New Zealand

**Keywords:** brain cancer, survival, elemental iron, ferrous iron, hypoxia, glioblastoma

## Abstract

**Introduction:**

Glioblastomas are an aggressive primary brain cancer, characterised by hypoxia and poor patient survival. Iron is the most abundant transition metal in the brain, yet data on the iron content of brain cancers is sparse. Ferrous iron is an essential cofactor for a super-family of enzymes, the iron- and 2-oxoglutarate-dependent dioxygenase enzymes (2-OGDD). These enzymes control the response to hypoxia via hydroxylation of the hypoxia-inducible factor-1α (HIF-1α), and DNA demethylation via hydroxylation of 5-methyl cytosines (5hmC).

**Methods:**

This study used clinical glioblastoma samples from 40 patients to determine the relationship between 2-OGDD activity and iron. Elemental iron was measured using inductively coupled plasma mass spectrometry (ICP-MS) and ferrous iron was measured using the colorimetric ferrozine assay. Iron measurements were compared against patient survival and clinicopathological data, and 2-OGDD-dependent activity of HIF-1 activation and 5hmC.

**Results and discussion:**

Elemental and ferrous iron levels were weakly related. Higher ferrous iron content of clinical glioblastoma tissue was associated with longer overall survival compared to lower ferrous iron content, but elemental iron showed no such relationship. Neither form of iron was related to clinicopathological data or markers of 2-OGDD activity. The impact of iron supplementation on the hypoxic response was assessed in three glioblastoma cell lines *in vitro*, similarly showing only a limited influence of iron on these 2-OGDD enzymes. Our data, together with prior studies in anaemic patients, highlight the importance of healthy iron levels in patients with glioblastoma, but further mechanistic studies are needed to elucidate the molecular pathways involved.

## 1 Introduction

Glioblastomas are highly aggressive primary brain cancers derived from glial cells located in the central nervous system (1, 2). Current clinical guidelines use isocitrate dehydrogenase (*IDH*) mutation status to separate glioblastoma (IDH wild type) from high-grade astrocytoma and oligodendrogliomas (IDH mutant) (3). Glioblastomas carry a poor prognosis, with a median survival of 15-31 months, and a high likelihood of recurrence (4, 5). Glioblastomas are particularly hypoxic which is related to their poor prognosis (6).

Iron is a vital micronutrient for biological processes in all cells, including for cell growth, oxygen transport, and metabolic processes (7). The ability of iron to reversibly convert from ferric iron (Fe^3+^) to ferrous iron (Fe^2+^) is essential for these biological processes (8, 9). Approximately 80% of the body’s iron stores is bound to the O_2_ transport proteins haemoglobin in erythrocytes and myoglobin in muscle. The remainder is either bound to Fe-containing haem proteins, such as peroxidases or cytochromes, non-haem iron proteins and enzymes, or stored in hepatocytes and macrophages and in the iron storage protein, ferritin (8, 9).

Ferrous iron is essential for a superfamily of enzymes, the iron- and 2-oxoglutarate-dependent dioxygenase enzymes (2-OGDD) (10–12). Besides iron, all 2-OGDD require ascorbate as cofactor, and molecular oxygen and 2-oxoglutarate as substrates. The human 2-OGDDs catalyse numerous hydroxylations, affecting vital biological mechanisms including DNA demethylation, cellular metabolism, and hypoxic adaptation (11, 12). The prolyl hydroxylases (PHD 1-3) and the asparagine hydroxylase factor-inhibiting hypoxia-inducible factor (FIH), control the hypoxic pathway by hydroxylating the hypoxia-inducible factor (HIF) regulatory α subunit, preventing activation of the HIF transcription factor (10, 13). The 2-OGDD enzyme family also includes enzymes that regulate gene expression. The ten-eleven-translocation enzymes (TET 1-3) hydroxylate 5-methyl cytosine (5mC) to 5-hydroxymethyl cytosine (5hmC) and further oxidation products, resulting in DNA demethylation and increased global gene expression (12, 14). Lysine and histidine demethylases that that modify histones, thus affecting global gene expression, are 2-OGDD family members (12, 15).

Iron has been investigated as a novel anti-tumour agent with respect to its capacity to induce ferroptosis, an iron-overload induced cell death (16), inhibition of cell migration (17), and promotion of anti-tumour immunity (18), among other potential functions. Macrophages, a major infiltrate in glioblastoma tumours, can store iron, especially under conditions of iron overload (19). Iron is the most abundant transition metal in the brain (20–22). Some regions within the brain, especially the iron-rich substantia nigra, caudate nucleus and globus pallidus, contain more iron than others (23). Of note, most gliomas arise in the frontal/temporal lobe, a region relatively low in iron. Astrocytes function as iron sensors to regulate and communicate the iron requirement of the brain via secreting hepcidin, which modulates the expression of iron regulatory proteins (9, 19, 24). Iron uptake into the brain is tightly regulated by endothelial cells and neighbouring astrocytes at the blood brain barrier (19, 24–26).

The rational above indicates that iron levels could exert a significant impact on cancer cell biology, yet it remains unclear whether iron content varies by location in the brain, or patient sex or age. The level of iron in glioblastoma tissue, and whether it impacts patient outcome, is unknown. It is not known whether iron plays a role in supporting 2-OGDD enzyme activity in glioblastoma cells. Our study aims to address these gaps in our knowledge. We have used clinical glioblastoma samples to measure iron content in tissues and determine associations with patient or tumour characteristics, and used glioblastoma cell lines grown in culture to ascertain the impact of iron levels on the activation of the hypoxic response.

## 2 Materials and methods

### 2.1 Materials

Unless stated explicitly, all chemicals were from Sigma-Aldrich (St Louis, MO, USA).

### 2.2 Human ethics, donor consent and glioblastoma samples

Most samples from this patient cohort have previously been analysed for ascorbate, IDH mutation status, hypoxic pathway activity and epigenetic analysis (27, 28). Human ethical approvals were from the University of Otago Ethics Committee (H19/163) and the national Health and Disability Ethics Committee (MEC/08/02/016), with approval for the use of samples from the Canterbury Tissue Bank Board (2001DPVR). Each donor provided written consent for the use of their sample and access to medical notes for research. After resection, samples excess to diagnosis were gifted to He Taonga Tapu Cancer Society Tissue Bank Christchurch or University of Otago Dunedin, and snap frozen (collection 2003-2019). Medical notes and pathology reports provided patient and tumour characteristics, as well as follow-up data (up to 2021). Consultation with Māori, Aotearoa/New Zealand’s indigenous people, was carried out through the University of Otago, with an option of sample disposal with karakia (blessing) offered to donors.

### 2.3 Glioblastoma sample processing

For ICP-Q-MS, clinical samples were cracked into small pieces in a pre-chilled mortar on dry ice, and carefully weighed. For the ferrozine assay, frozen tissue samples were placed into an Eppendorf tube with potassium phosphate buffer (pH 7.4) and homogenized using an Eppendorf micro-pestle. Following centrifugation, supernatant was used for the ferrozine assay.

### 2.4 Inductively coupled plasma-quadrupole-mass spectrometry

All chemicals were specifically ultra-trace analysis grade. After thawing, samples were digested in 14 N nitric acid and moved to precleaned perfluoroalkoxy-polymer (PFA) vessels (Savillex, USA) for digestion preparation. The PFA vessels were capped, and the acid reflux process was completed overnight at 105°C to completely digest the tissue samples. Subsequently, the solution was allowed to evaporate to dryness, followed by addition of 50 µL of hydrogen peroxide (30% vol/vol) and a further 50 µL of 14 N nitric acid to complete digestion of samples. Once digested and dried, 3 mL of 2% nitric acid (vol/vol) was added to the PFA vessel, the mixture was warmed to complete the solubilization process before being transferred into auto-sampler vials for ICP-Q-MS analysis. The concentrations of iron, sodium, magnesium, phosphorus, potassium, calcium, copper, and zinc in the glioblastoma tissue samples were measured using an Agilent 7900 ICP-Q-MS (Agilent, Santa Clara, USA). Predetermined cocktails of standard elements were used as internal controls and for quality assurance and standardization of the instrument; a calibration against serially diluted National Institute of Standards and Technology traceable standards was used. Elements of interest were measured in standards and samples with a helium collision gas tune to eliminate polyatomic interferences. To determine effect of extraction on the ICP-Q-MS measurements, an extraction blank was measured on five empty cryovials, and the average mass of each element of interest was subtracted from the samples. The density of solutions was known and the concentration of elements in the glioblastoma tissues was calculated using known volumes and the tissue weights that were initially recorded.

### 2.5 Ferrozine assay

Ferrous iron was measured as previously described (29, 30). Frozen tissue samples were placed into an Eppendorf and homogenized on ice using an Eppendorf micro-pestle with 200 µL of ice-cold potassium phosphate buffer (pH 7.4). The sample was centrifuged at 10,000 rcf for 10 minutes at 4°C and 200 μL of the supernatant was added to 600 µL of ferrozine stock (4.6 mM sodium L-ascorbate, 17mM sodium acetate, 0.18 mM ferrozine (3-(2-pyridyl)-5,6-diphenyl-1,2,4-triazone 4’,4”-disulfonic acid sodium salt, Cat no 82950)). A standard curve was created using FeSO_4_ (0.5 µg/mL to 100 µg/mL in 0.01 N HCl; AnalR BDH Cat no 10112) (**Supplementary Figure 1**). The samples were measured at 550 nm using a Wallace 1420 Victor microplate reader (PerkinElmer Life and Analytical Sciences, USA). The ferrous iron concentrations of the glioblastoma samples were interpolated from the standard curve.

### 2.6 Cell culture

Human glioblastoma cells (U251MG, T98G, U87MG) were sourced from the European Collection of Authenticated Cell Cultures (ECACC, Sigma-Aldrich, St Louis, MO, USA). Cells were grown in minimum essential media (MEM, Thermo Fisher Scientific, Waltham, MA USA) substituted with 10% foetal bovine serum (FBS, Gibco, Thermo Fisher), 1% sodium pyruvate and 1% non-essential amino acids with 1% penicillin/streptomycin. Cells were maintained in a 37°C humidified incubator in air with 5% CO_2_, used at low passages (<20), and confirmed negative for mycoplasma (e-Myco™ Mycoplasma PCR Detection Kit, iNtRON Biotechnology, Korea).

### 2.7 Iron manipulation *in vitro*

Glioblastoma cells were exposed to ferrous sulphate (100 μM FeSO_4_, made fresh at 10 mM stock in MEM). Cells were either exposed to FeSO_4_ for 24 h in normoxia (air with 5% CO_2_), followed by exposure to normoxia or mild hypoxia for 24 h (5% O_2_, 5% CO_2_, balance N_2_, Xvivo System Model X3, BioSpherix). Cells were collected for western blotting.

### 2.8 Western blotting of *in vitro* samples

Western blotting was carried out as previously described (31). Following treatments, cells were lysed in samples buffer (60 mM Tris pH 4.8, 2% SDS, 20% glycerol, 0.1 M 1,4 dithiothreitol (DTT) with 1x cOmplete™ protease inhibitors and 0.02% bromophenol blue) for Western blot analysis. Proteins from the cell lysates were separated by SDS-PAGE using 4-12% gradient Bis-Tris Plus SDS gels (Invitrogen, Thermo Fisher, Auckland NZ) and transferred to polyvinylidene difluoride membranes. Proteins of interest were detected using primary anti-human antibodies (HIF-1α (1:250, 610959, BD Transduction Laboratories, USA), Bcl2-interacting protein 3 (BNIP3, 1:1000, AF4147, R&D Systems, Invitro Technologies, Auckland, NZ), carbonic anhydrase 9 (CA-IX, 1:1000, AF2188, R&D Systems, Invitro Technologies, Auckland, NZ), β-actin (1:10,000, A5316)), with suitable secondary antibodies and ECL Select Western Blotting Detection Reagent (GEHERPN2235, Bio-Strategy, Auckland, NZ). Proteins of interest were detected using the Alliance Q9 imaging software and measured using the Alliance Q9 software, with β-actin as loading control.

### 2.9 Statistics

Data were analysed using GraphPad Prism (V10) with significance set at p<0.05. The Schapiro-Wilks test was used to test normality, Mann-Whitney or unpaired t-tests to test associations, and paired t-test was used to compare means between FeSO_4_-treated and untreated groups. Spearman’s and Pearson’s were used to test correlations. Kaplan-Meier survival curves were analysed using Log rank tests. Cell culture experiments included ≥3 independent repeats.

## 3 Results

Glioblastoma samples from a total of 40 patients were available for analysis (**Table 1**). Elemental iron was measured in all samples, whereas ferrous iron could only be measured in 39 of the samples (**Table 1**). Most patients identified as European with only 2 identifying as Māori or Pacific peoples, and most were male with a median age of 59 years (**Table 1**). Although all tumours were initially described as glioblastoma, three were subsequently redefined as high-grade astrocytoma according to their IDH^R132H^ mutation status. Similar numbers of tumours were located in the frontal, temporal and parietal lobes, with few located in the occipital part of the brain (**Table 1**). Analyses of ascorbate, hypoxic pathway and epigenetic levels of most of this cohort have been published (27, 28).

**Table 1:** Patient and tumour characteristics.

Parameter	Number (%)
total	40 (100)
Iron data	
ICP-Q-MS	40 (100)
Ferrozine	39 (98)
Sex	
Female	10 (25)
Male	24 (60)
not available	6 (15)
Age at diagnosis	
<60 years	13 (32)
≥60 years	21 (53)
not available	6 (15)
Ethnicity	
Maori/Pacifica	2 (5)
European	28 (70)
Other	4 (10)
not available	6 (15)
IDH ^R132H^	
Glioblastoma, IDH wild type	37 (93)
Astrocytoma grade IV, IDH mutant	3 (7)
Tumour location	
Frontal	13 (32)
Temporal	11 (28)
Parietal	8 (20)
Occipital	2 (5)
not available	6 (15)

**Figure 1:**
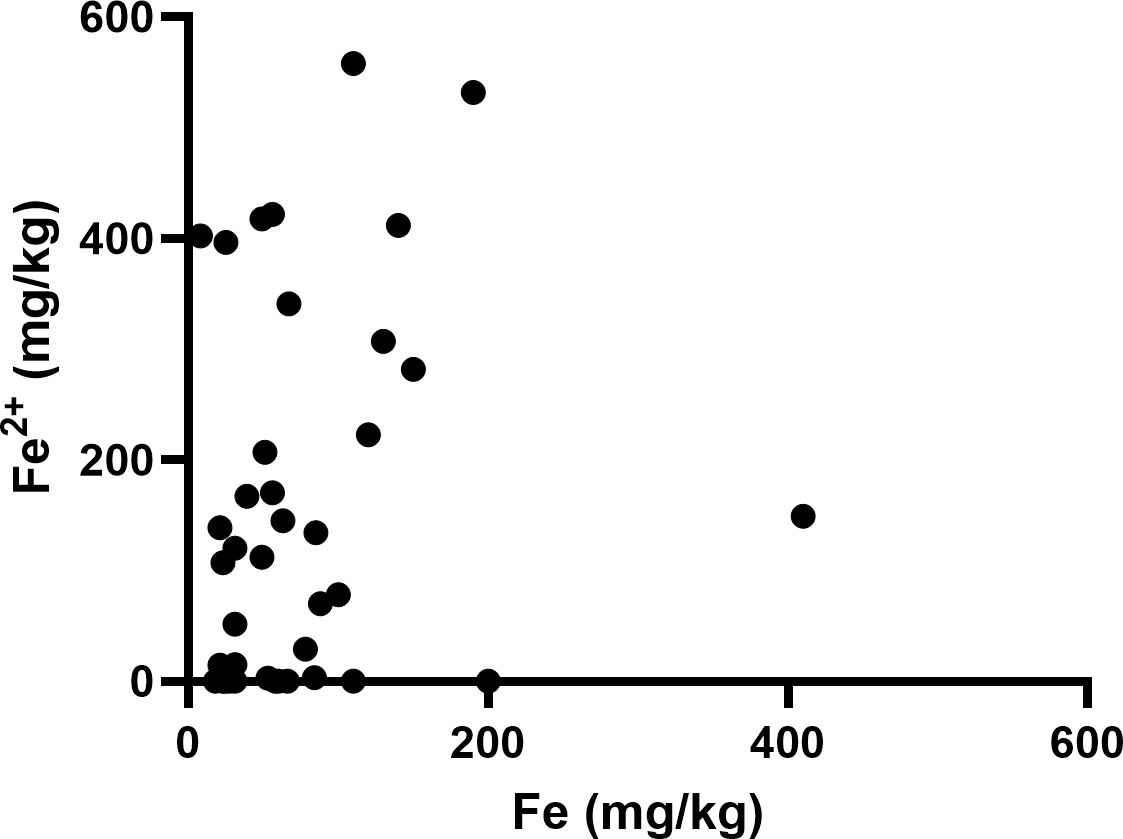
Iron content of glioblastoma samples. Correlation between ferrous iron measured by ferrozine assay and elemental iron measured by ICP-MS (Spearman’s correlation r=0.26, p=0.1, n=40).

**Figure 2:**
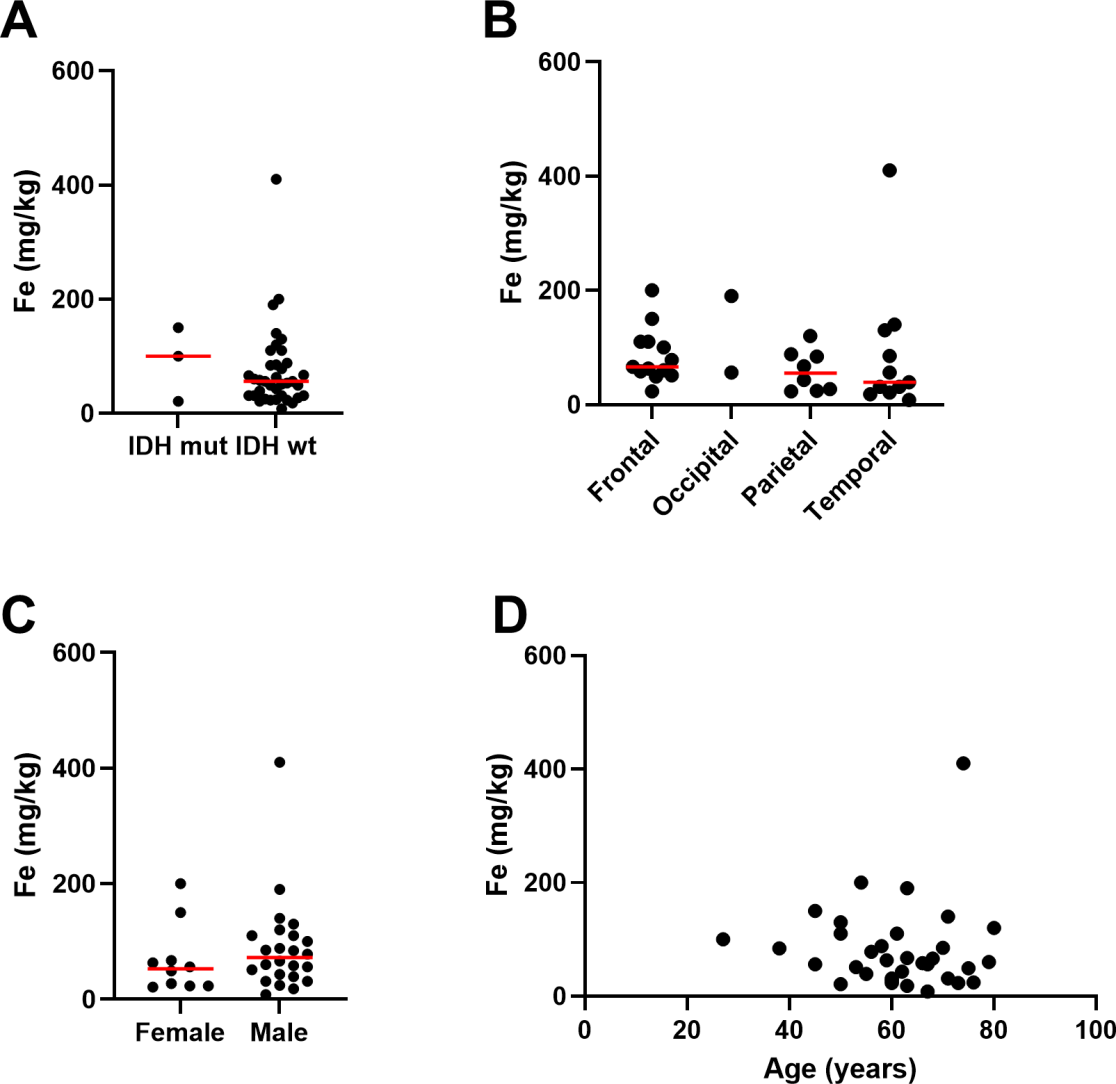
Associations between elemental iron and clinicopathological data. Associations between iron measured by ICP-Q-MS in glioblastoma samples and **(A)** IDH mutation status of glioblastoma (Mann Whitney p=0.63, n=40), **(B)** location of tumour in the brain (Kruskal-Wallis p=0.36, n=34), **(C)** sex of patient (Mann Whitney p=0.31, female n=10, male n=24), and **(D)** age of patient (Spearman’s correlation r-0.15, n=34). Median is shown as red line.

**Figure 3:**
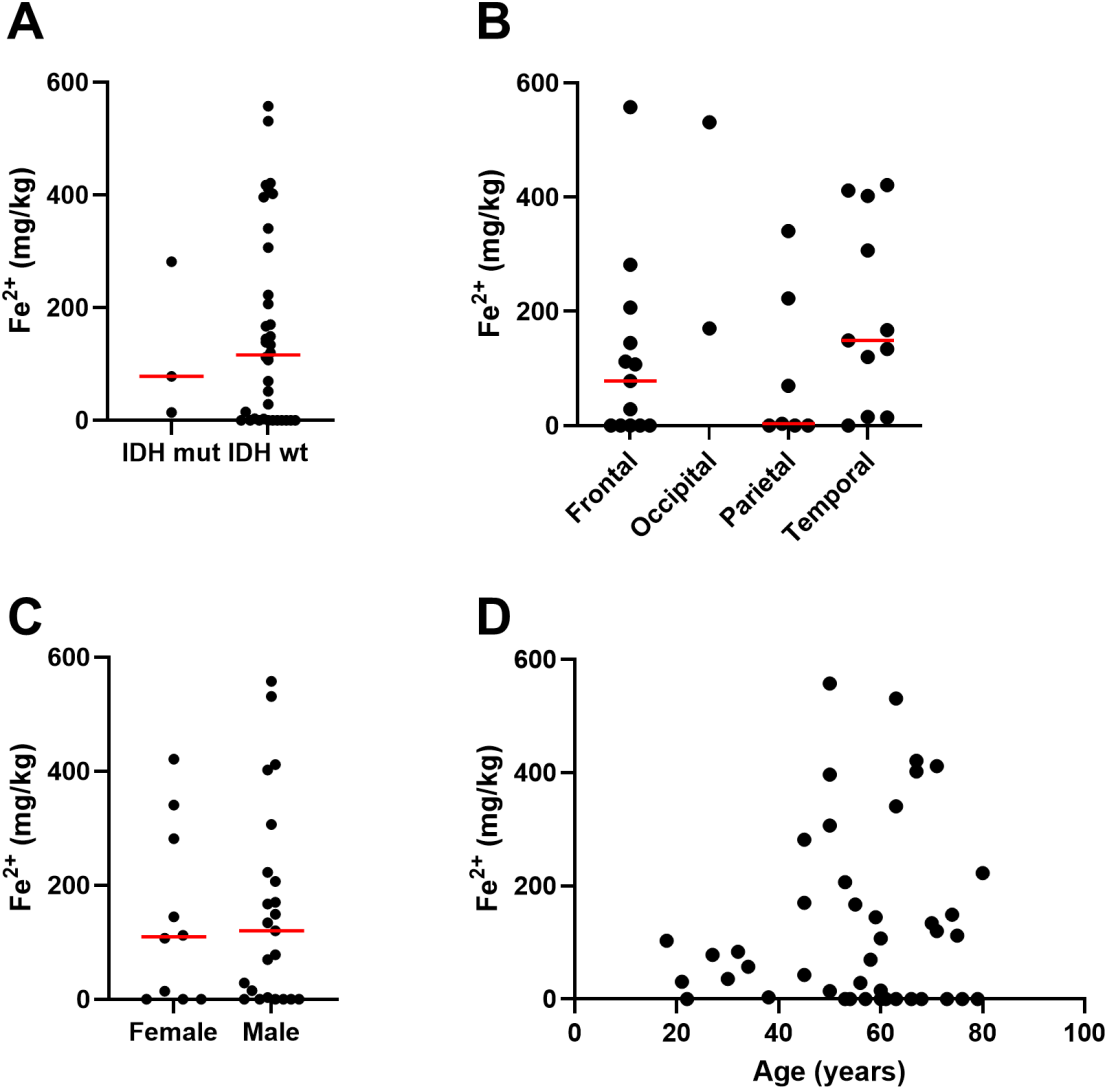
Associations between ferrous iron and clinicopathological data. Associations between iron measured by the ferrozine assay in glioblastoma samples and **(A)** IDH mutation status of glioblastoma (Mann Whitney p=0.97, n=39), **(B)** location of tumour in the brain (Kruskal-Wallis p=0.16, n=31), **(C)** sex of patient (Mann Whitney p=0.83, female n=10, male n=23), and **(D)** age of patient (Spearman’s correlation r 0.03, n=29). Median shown as red line.

Elemental iron was measured by ICP-Q-MS, showing a median of 57 mg/kg and a mean of 75 ± 72 mg/kg (**Table 2**). Samples contained between 2.5 – 200 mg/kg of elemental iron, with one very high measurement (410 mg/kg, **Figure 1**). Ferrous iron was estimated using the ferrozine assay, showing a median 112 mg/kg and a mean of 154 ± 169 mg/kg, ranging from 0 to 558 mg/kg (**Figure 1**). There was only a weak correlation between elemental and ferrous iron (**Figure 1**, r=0.26, p=0.1). Elemental iron content was similar in IDH mutant and IDH wild type tumours, and did not vary significantly by tumour location, patient sex or patient age (**Figure 2**). Similarly, ferrous iron content did not vary significantly by IDH mutation status, location, sex or age (**Figure 3**).

**Table 2:** Measurement of chemical elements in glioblastomas.

Parameter	Median	Mean	SD	Detection Limit*	Samples n=
ICP-Q-MS (mg/kg)					
Fe	57.0	75.4	72	1.5	40
Na	2200	2238	736	15.0	40
Mg	100.0	92.8	43	3.0	40
P	1400	1364	550	15.0	40
K	1350	1533	912	15.0	40
Ca	84.0	116.1	113	30.0	40
Cu	1.9	2.3	1.6	0.0	40
Zn	12.0	13.8	5.3	9.0	40
Ferrozine (mg/kg)					
Fe^2+^	112.3	154.0	168.7		39

*Detection Limit based on 10 mg of sample.

**Figure 4:**
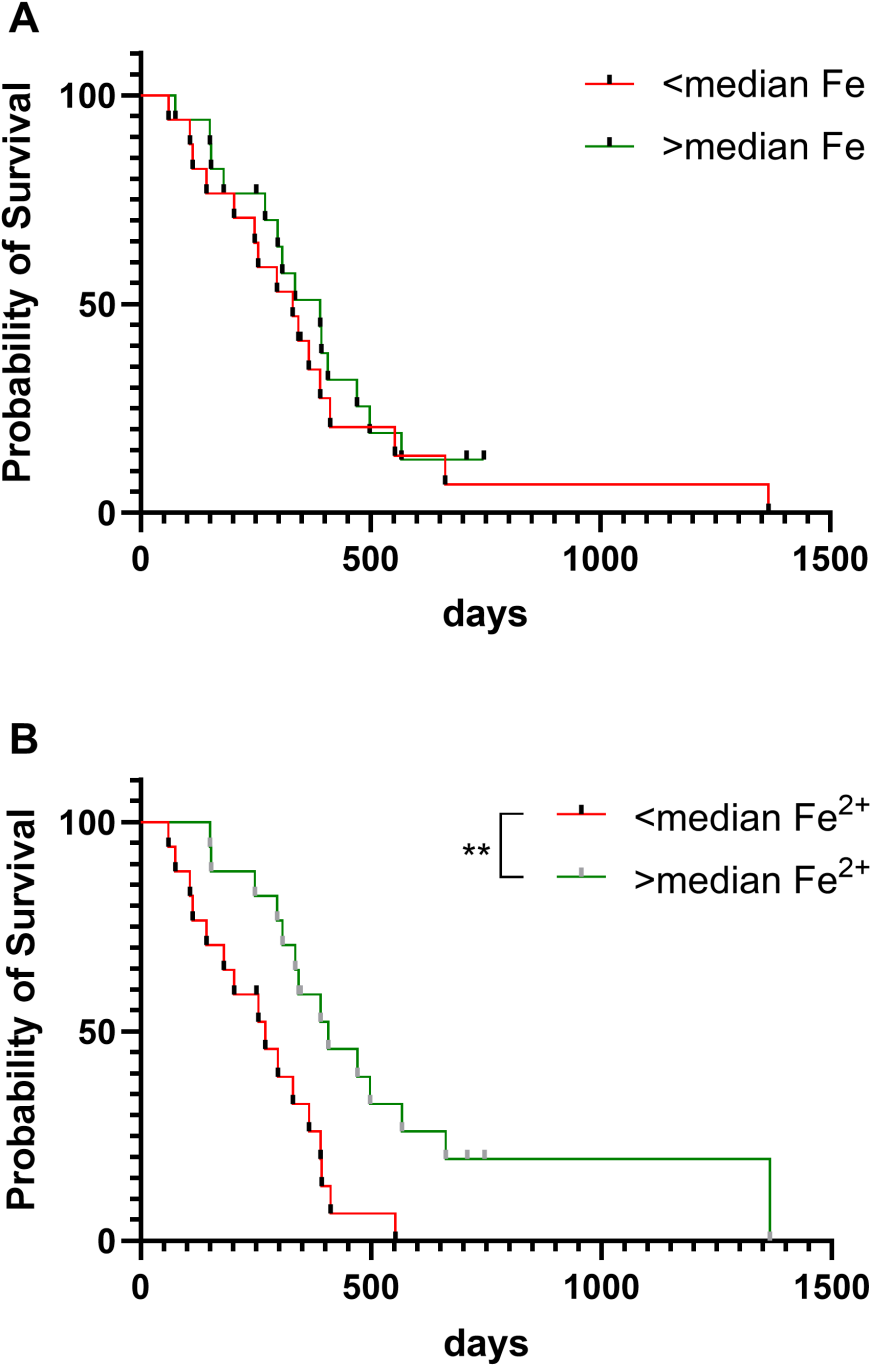
Patient survival according to iron content of glioblastoma tumours. Overall survival of patients with glioblastoma according to **(A)** elemental iron content of tumours (Log-rank test, p=0.50, n=34), and **(B)** ferrous iron of tumours (Log-rank p=0.005, n=33). Samples were divided into two groups of below or above median elemental iron (61.5 mg/kg) or median ferrous iron (112 mg/kg). ** p < 0.01.

We next determined whether iron content of glioblastoma samples was associated with patient outcome (**Figure 4**). Overall survival of patients with glioblastoma was associated with ferrous iron availability: survival was significantly shorter for those patients with glioblastoma tumours that contained below median ferrous iron, compared to those with above median ferrous iron content (**Figure 4B**). In contrast, survival did not vary by median elemental iron content (**Figure 4A**).

Other chemicals were measured by ICP-Q-MS in glioblastoma samples at the time of measuring elemental iron. Glioblastomas contained the following median levels: sodium (2200 mg/kg); phosphate (1400 mg/kg); potassium (1350 mg/kg); magnesium (100 mg/kg); calcium (84 mg/kg); copper (1.9 mg/kg) and zinc (12.0 mg/kg) (**Table 2**). We then compared data from glioblastoma samples in our study to two previously published studies that used ICP-MS to quantify elements in different regions of human brain (**Table 3**). Due to large variations between individual samples, there appeared to be no major difference between elements (Fe, Na, Mg, P, Ca, Cu, Zn) in glioblastoma tissue and regions in non-cancerous brain (iron-rich globus pallidus and caudate nucleus, hippocampus) (**Table 3**).

**Table 3:** Comparison of elements measured in brain tissue.

	Current study	South African study ^a^			Austrian study ^b^	
Parameter (mg/kg)	Glioblastoma	Globus pallidus	Caudate nucleus	Hippo-campus	Globus pallidus	Caudate nucleus
Fe	75 ± 72	103 ± 32	47 ± 18	24 ± 12	203 ± 38	106 ± 26
Na	2238 ± 736	3422 ± 374	3310 ± 354	3441 ± 380		
Mg	93 ± 43	24 ± 10	21 ± 7	21 ± 6	110 ± 34	95 ± 19
P	1364 ± 550	3348 ± 450	2755 ± 328	3046 ± 385		
Ca	116 ± 113	134 ± 72	119 ± 64	111 ± 27	64 ± 14	73 ± 13
Cu	2.3 ± 1.6	4 ± 1	2 ± 1	2 ± 1	7 ± 2	6 ± 1
Zn	13.8 ± 5.3	8 ± 23	6 ± 3	6 ± 8	13 ± 3	12 ± 3

Our study glioblastoma n= 40, South African n= 42, Austrian n= 11; mean ± SD; ^a^ (34), ^b^ (33).

**Figure 5:**
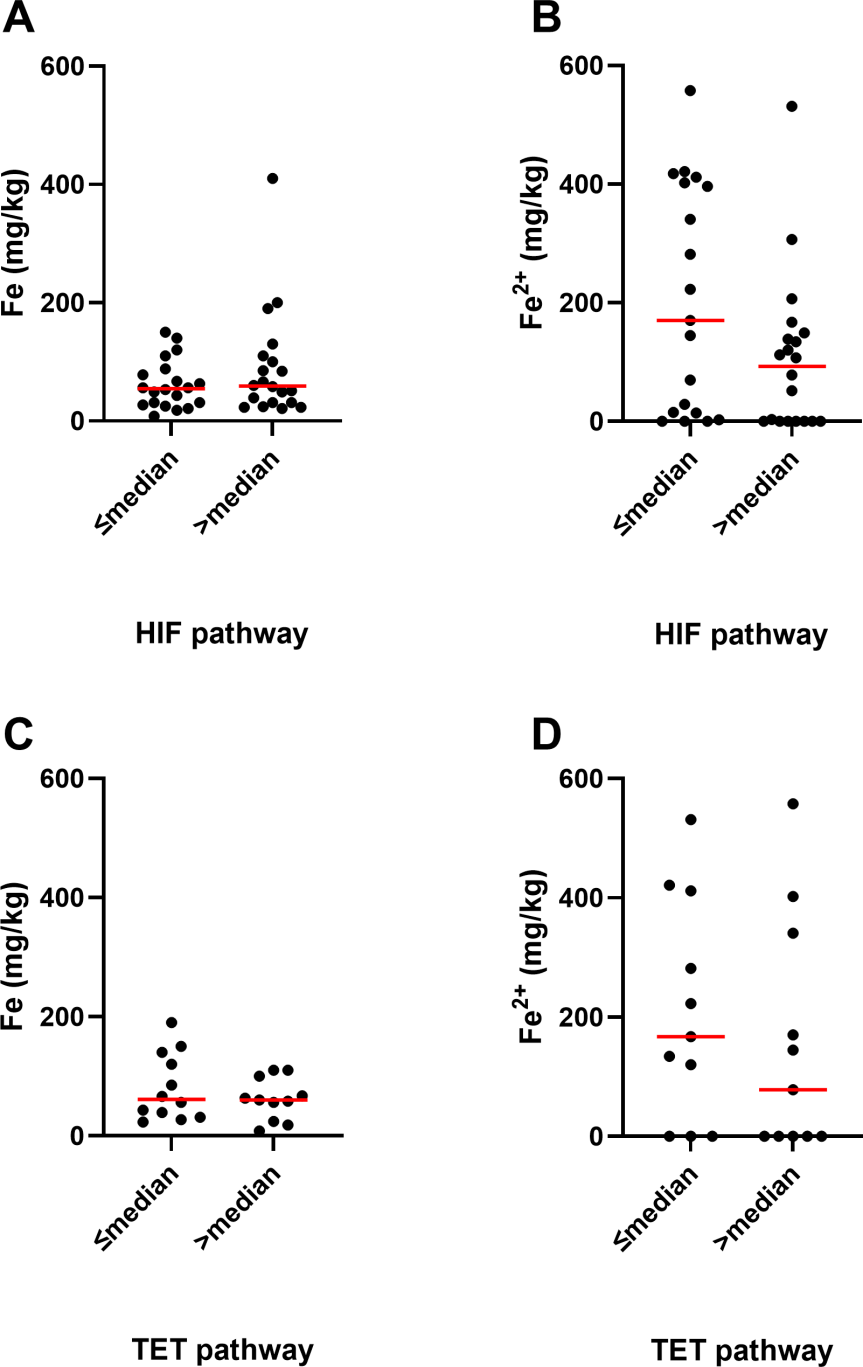
Association between iron and activity of 2-OGDDs in glioblastoma samples. Association between elemental iron and **(A)** above or below median relative HIF pathway (p=0.50, n=40), and **(C)** above or below median TET pathway (p=0.54, n=23). Association between ferrous iron and **(B)** above or below median relative HIF pathway (p=0.08, n=39), and **(D)** above or below median TET pathway (p=0.44, n=22). Associations were tested using Mann Whitney test. The median relative HIF pathway score was 2, and the median TET pathway score was 0.18% 5hmC. Median shown as red line.

As ferrous iron is an integral part of the active site of all 2-OGDD enzymes, we investigated whether iron content was associated with 2-OGDD enzyme activity. This was done by analysing the relationships of elemental iron or ferrous iron content of glioblastoma samples with the relative HIF-score, as a measure of PHD and FIH activity (27), or percentage 5hmC, as a measure of TET activity (28) (**Figure 5**). The relative HIF-score was previously derived from protein data by combining HIF-1α and 6 HIF-regulated proteins into a single relative score for each glioblastoma sample (27). Similarly, the 5hmC data was previously measured using mass spectrometry in a selection of the glioblastoma samples and reported (28). The HIF-score was not significantly associated with elemental iron (p=0.50, **Figure 5A**), or ferrous iron (p=0.08, **Figure 5B**). Similarly, 5hmC data was not associated with either elemental or ferrous iron (p=0.54 and p=0.44, respectively, **Figures 5C, D**). This indicated no clear relationship between iron content and 2-OGDD enzyme activity in clinical glioblastoma samples.

**Figure 6:**
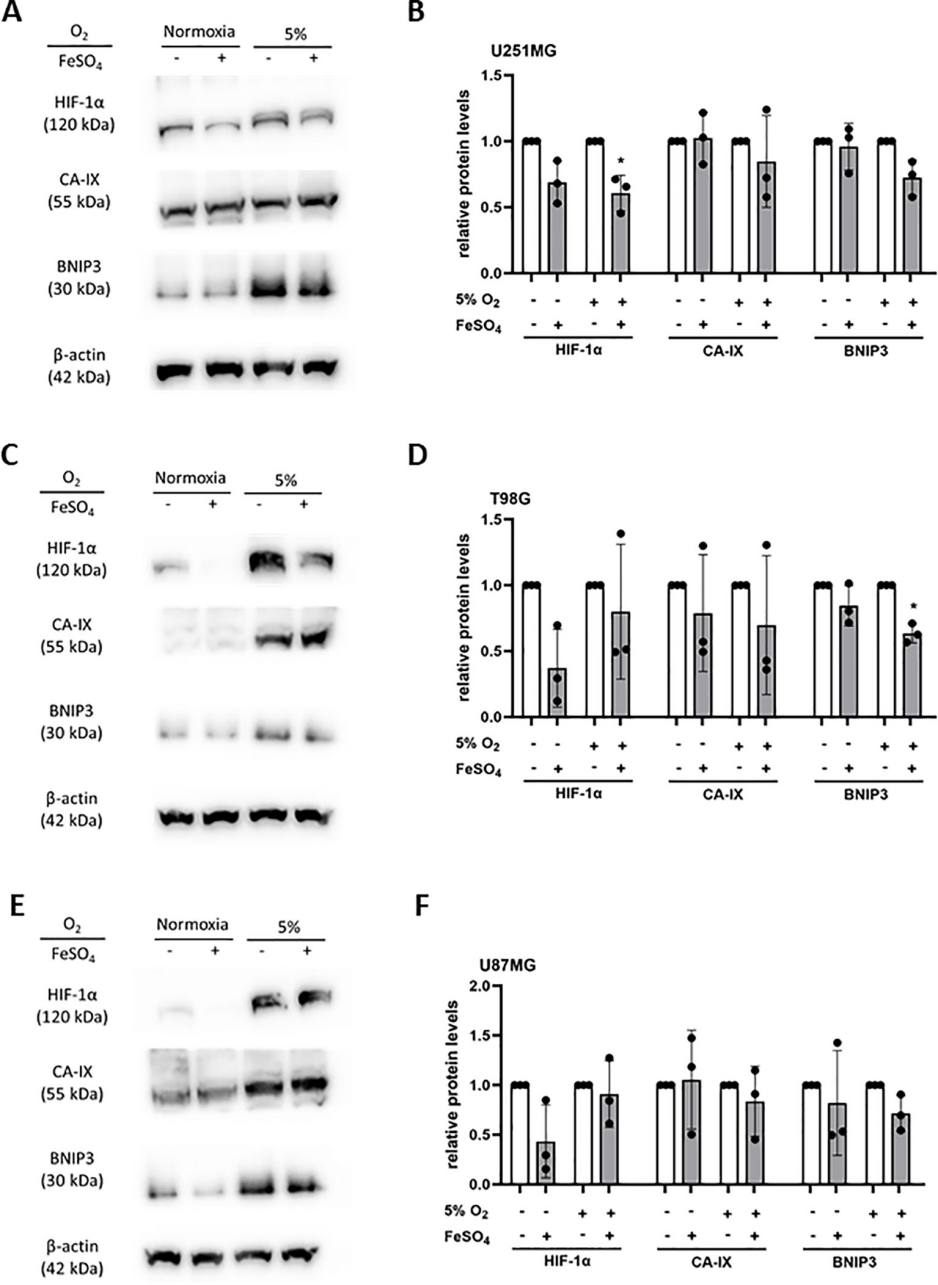
Hypoxic pathway response of glioblastoma cells exposed to iron supplementation. Cells were exposed to FeSO_4_ (100 μM) in normoxia, or exposed to FeSO_4_ in normoxia, then incubated in mild hypoxia (5% O_2_). Lysates of **(A)** U251MG, **(C)** T98G and **(E)** U87MG were collected for western blotting. Relative levels of HIF-1α, CA-IX and BNIP3 were used to analyse the hypoxic pathway, with β-actin used as loading control. Protein bands on western blots were quantified for relative protein levels in **(B)** U251MG, **(D)** T98G and **(F)** U87MG cells (with highest expression set as 1). Paired t-test; mean ± SD; n=3; * p<0.05.

To further explore the role of iron in 2-OGDD enzyme activity, we studied glioblastoma cells *in vitro*. Three glioblastoma cell lines (U251MG, T98G, U87MG) were exposed to FeSO_4_ with or without mild hypoxia (5% O_2_), and their hypoxic pathway response was analysed (**Figure 6**). In response to hypoxia, all three glioblastoma cell lines showed a robust stabilization of HIF-1α and an increase in BNIP3 protein, with T98G and U87MG also exhibiting an increase in CA-IX protein (**Figures 6A, C, E**). The addition of ferrous iron significantly reduced hypoxia-induced HIF-1α in U251MG cells and BNIP3 in T98G cells (**Figures 6B, D**), and did not significantly or consistently suppress the hypoxic pathway *in vitro* (**Figures 6B, D, F**). This suggests that the addition of ferrous iron did not affect the activity of the 2-OGDD enzymes PHD and FIH in glioblastoma cells *in vitro*.

## 4 Discussion

Elemental and ferrous iron content varied across clinical glioblastoma samples. There were no apparent associations between iron content and clinicopathological data. However, higher ferrous iron content of tumours was associated with improved overall survival of patients with glioblastoma. The iron content of clinical samples was not associated with 2-OGDD enzyme activity, and the lack of association between ferrous iron and the hypoxic response was confirmed *in vitro*.

Elemental and ferrous iron correlated only weakly, with Fe^2+^ appearing to be at a higher concentration than Fe. As elemental iron should encompass all forms of iron, irrespective of ionization, this was unexpected. Both ICP-Q-MS and ferrozine methods have been described as sensitive and accurate (29, 32). To our knowledge, ferrous iron has never been measured using the ferrozine assay in glioblastoma tissue. Our data was similar to elemental iron content reported for non-cancerous brain (33, 34). We did not have access to normal, uninvolved brain tissue from our patient cohort as that is not routinely resected during brain cancer surgery in New Zealand. The previously reported data was obtained from formalin-fixed tissue post-mortem analysed by MCP-MS (33), compared to our fresh-frozen tissue; how this may affect the comparative data is unclear.

A variety of techniques are used to measure iron within the brain, including X-ray fluorescence and emission iron mapping, magnetic resonance imaging (MRI), matrix-assisted laser desorption/ionization mass spectrometry, and inductively coupled plasma mass spectrometry (ICP-MS) (33–38), with very few reporting iron content of glioma tumours. In an MRI imaging study, 59 patients with glioma were scanned and quantitative susceptibility maps were constructed that enable estimation of the concentration of iron in an area of tissue (39). The study found that the mean basal ganglia susceptibility, or iron content, increased with glioma grade (39). X-ray fluorescence has been used to estimate iron content in glioma, but data was inconclusive due to small cohorts (40, 41).

Only one study has previously measured elemental iron in brain tumour tissue samples using ICP-MS (42). The study included tumour tissue (n=22, including n=7 glioblastoma and n=8 meningioma) and non-tumour brain tissue from a subset of the patients (n=6), and suggested that non-tumour brain tissue tended to have higher median iron content than tumour tissue, a finding we could not test. A study using X-ray fluorescence to measure a range of trace elements in both gliomas and normal brain tissue, reported that lower concentrations of iron were present in the glioma tissue when compared to the normal brain (40). The discrepancies may be influenced by the regions of brain that were sampled, as different regions are known to vary in iron content (20, 21, 23), and specifically, which brain region the glioma originated from. We saw no clear association between iron content and brain region of the tumours. Patients with glioblastoma may develop aplastic anaemia, especially after treatment with temozolomide, a standard treatment for patients with glioblastoma (43). This would influence iron measurements of brain tumours; however, our samples were all from patients prior to treatment. The blood iron status of our patients was not determined, and may have influenced tissue content, and account for the large inter-individual variations.

Follow-up data from the patient cohort showed a striking association between ferrous iron content of glioblastoma tissue and overall patient survival, although elemental iron appeared to be unrelated to outcome. The reason for the association with ferrous iron is unclear. Our previous study, that included all patients with glioblastoma in this study, had shown a significant association between poor overall survival and an activated hypoxic pathway (27). However, as our current data shows little or no relationship between ferrous iron and the hypoxic pathway, this association with patient survival was unexpected.

We propose that tissue levels of ferrous iron reflect global iron status, such that low tissue levels may indicate anaemia. As anaemia information is not available for our glioblastoma cohort, we were unable to determine associations between tissue iron levels and blood status. Anaemia in patients with glioblastoma has been associated with poor survival in most (44–48), but not all studies (49). A recent study reported that anaemia was associated with poor survival predominantly in male, but not female, patients with glioblastoma (48). Our survival data was not driven by sex bias, as both cohorts (above and below median ferrous iron) had similar male/female ratios. Anaemia, reflected in reduced haemoglobin or haematocrit readings (48), is associated with hypoxemia, a negative prognostic marker for glioblastoma (6). Whether there are subtle differences between hypoxemia, a reduction in blood oxygenation, and hypoxia, a localised drop in tissue oxygen levels, and their effect on the HIF-pathway is unclear.

We hypothesised that the concentrations of iron in glioblastoma tissue would correlate with 2-OGDD activity. These enzymes require ferrous iron and ascorbate as cofactors (11, 12), and we have previously detected strong correlations between tissue ascorbate content and 2-OGDD activity in glioblastoma (27, 28). The enzyme family include the HIF hydroxylases (PHDs and FIH) and TETs, with TETs having among the highest K_m_ for iron of the known 2-OGDDs, ten to 100-fold above the HIF-hydroxylases (11, 50). This would imply that a reduction in iron could specifically reduce TET activity, leading to lower 5hmC levels. Yet, we saw no relationship between 5hmC levels and iron content in clinical glioblastoma samples. Interestingly, cancer-associated TET mutants display markedly lower K_m_ values towards iron, compared to non-mutated TET enzymes (50), suggesting tighter iron binding by these enzymes and a reduced dependency on an extraneous iron source. Previous data had shown that the activity of TET enzymes was reduced in glioma compared to normal brain (51), and that decreased TET activity and lower 5hmC were associated with poor patient prognosis (52, 53).

Previous *in vitro* studies have shown that iron chelation (DFO) or iron displacement (NiCl_2_ or CoCl_2_) resulted in the suppression of 2-OGDD activity, resulting in decreased HIF-hydroxylase activity, with subsequent increased levels of HIF-1α and its downstream targets (31, 54). These reagents poison the enzyme active site by removal or displacement of the active ferrous iron (31, 55). We had therefore hypothesized that an increased iron supply would be associated with increased hydroxylase enzyme activity, but neither our clinical nor *in vitro* data supported this hypothesis.

The brain is known to have a high iron content (21, 22), and therefore iron may not be a limiting factor for 2-OGDD activity, unlike ascorbate (27, 28). We confirmed this lack of association between iron and 2-OGDD activity in glioblastoma cells in culture, where supplementation with FeSO_4_ did not consistently suppress the hypoxic pathway. Increasing iron concentration *in vitro* was challenging, because standard cell culture medium contains 10% bovine serum, that may provide additional iron. It is therefore plausible that both clinical tissues and cells in culture have sufficient bioavailable (intracellular) ferrous iron for the activity of 2-OGDDs.

This study has focused on high-grade primary brain cancer, and it remains to be investigated whether lower-grade primary brain cancers (28), or brain metastases (56), have similar iron content, or relationship between ferrous iron and 2-OGDD, or patient survival.

The main limitation of our study, besides a relatively small cohort size, is that iron-related proteins were not assessed. Information on transferrin, ferroportin, ferroreductase, transferrin receptor, ferritin and others would have provided a more complete picture of the iron pathways in glioblastoma tumours. In addition, further mechanistic studies in serum-free cell culture are required to confirm the role ferrous iron for 2-OGDD activity, specifically for TET activity.

In conclusion, iron content varied greatly between individuals, and ferrous iron content of glioblastoma tumours may be associated with patient outcome, but iron appears not to be a limiting factor for 2-OGDD activities in glioblastoma clinical samples or cell culture. Our data, together with prior studies in anaemic patients, highlight the importance of healthy iron levels in patients with glioblastoma, but further mechanistic studies will be needed to elucidate the molecular pathways involved.

## Data Availability

The original contributions presented in the study are included in the article/**Supplementary Material**. Further inquiries can be directed to the corresponding author.
