# Supplementary material for: Iron content of glioblastoma tumours and role of ferrous iron in the hypoxic response in vitro
Source: Front Oncol. 2025 Mar 7;15:1536549. doi: 10.3389/fonc.2025.1536549 (PMC11925887; doi:10.3389/fonc.2025.1536549)
Supplement: Supplementary file 1 [file DataSheet1.pdf]

Iron content of glioma tumours and role of ferrous iron in the hypoxic response *in vitro*

Citra Praditi[1#], Eira Beverley-Stone[1#], Malcolm Reid[2], Eleanor Burgess[1], Rebekah L Crake[3], Margreet CM Vissers[4], Janice A Royds[5], Tania L Slatter[5], Gabi U Dachs[1*], Elisabeth Phillips[1]

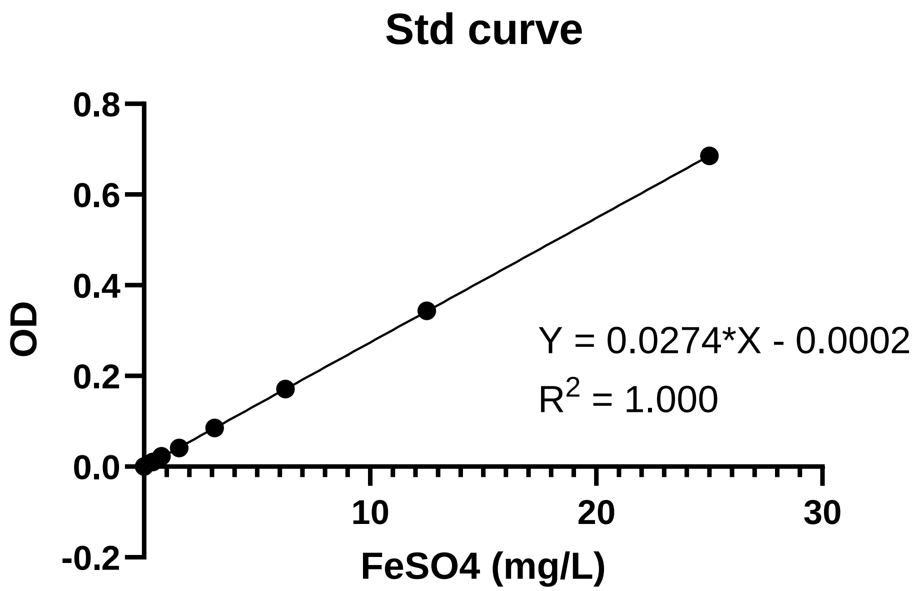

Supplementary Figure 1 Standard curve for the measurement of ferrous iron using ferrozine, with linear regression equation.
